# The Army Pharmaceutical Service

**Published:** 1915-04-24

**Authors:** 


### THE ARMY PHARMACEUTICAL SERVICE.

Since the beginning of the war many complaints have been heard about the ungenerous treatment accorded to Army dispensers. It is pointed out that the authorities do not exercise adequate discrimination between trained and qualified men and untrained men; that the pay is hopelessly disproportionate to the proper qualification and duties; and that the dispenser has no status at all or, at the best, a very inferior one. A close scrutiny into the matter makes it clear that of these three grievances the last two are dependent upon the first, and if the ground for that could be removed a very important step would be taken in the direction which dispensers consider to be the right one. If a person without any statutory qualification is allowed to act as a dispenser it is not altogether desirable that the position should carry with it greatly improved status and more substantial pay. On the other hand, if it were required of an Army dispenser that he should have undergone the orthodox training of a dispenser in civil life and passed the usual tests, the case for better treatment would be considerably strengthened. Under the conditions now ruling in our own Army it is of little use to point to the Pharmaceutical Services of the Armies of our Allies and attempt to draw the inference that our dispensers should have commissioned rank because that position is accorded to pharmacists in the French and Russian Armies. In France—and the same is the case, no doubt, in Russia—the military pharmacist is recruited solely from the ranks of duly qualified pharmacists; but, as is common knowledge, that is not so in this country. The first step, then, would be to persuade the authorities here that their present system is fundamentally wrong, and that it is necessary to reorganise the whole of this part of the Army Medical Service. Attempts to do this in time of peace have not been successful, and it is, therefore, at least doubtful whether in time of war those responsible for the organisation of this service would be more favourably disposed to consider the claims of dispensers than they were at a time when they had more leisure to deal with questions of this kind. And yet qualified dispensers have, unquestionably, a good case; and, what is equally important, it seems to have been presented properly. It is, therefore, difficult to understand why the authorities cling to their old-fashioned method of organisation in regard to the Pharmaceutical Service when it could so easily be improved. This fondness for tradition in the case of Army dispensing stands out in strong contrast when one considers the wonderful improvements that have been brought about in other directions. At a time like the present no loyal subject would wish to press the authorities unreasonably, but there can be no room for doubt that the Army Pharmaceutical Service could be organised on better lines than it is.

Pharmacists could be named already holding commissions in the Army—not in the Medical Service—who are admirably suited to the task of organising a Pharmaceutical Service much better than the present one; and there are certainly several who are not in the Army who could do it equally well. Moreover, dispensers have not desired to publish their grievances at a time when much greater problems confront those in authority. A few letters have appeared in one of the London papers, and it is satisfactory to note that, according to the writer of one of these, chemists as a body have not allowed "their inferior status to interfere with their duty to the nation in the hour of need." But it is unfortunate that an attempt should have been made in the public Press to use the anomalous position of the Army dispenser as a peg on which to hang a claim to put holders of the dispensers' certificate of the Society of Apothecaries on the same footing in civil life as duly qualified chemists and druggists. Such an argument as this can have nothing but a harmful influence on the dispenser's position *vis à vis* the military authorities.

In this issue of The Hospital is published a letter which deals with an interesting phase of the subject. A correspondent draws attention to the position of certificated dispensers now serving in military hospitals in place of the Regular R.A.M.C. compounders, and draws attention to the prevailing anomalous conditions. This is part of the greater question of the position of military dispensers generally, and the very genuine grievance that he writes about certainly ought to be remedied.

